# M2 Macrophages as a Potential Target for Antiatherosclerosis Treatment

**DOI:** 10.1155/2019/6724903

**Published:** 2019-02-21

**Authors:** Ying Bi, Jixiang Chen, Feng Hu, Jing Liu, Man Li, Lei Zhao

**Affiliations:** ^1^Department of Neurology, Union Hospital, Tongji Medical College, Huazhong University of Science and Technology, Wuhan 430022, China; ^2^Department of Dermatology, Wuhan No.1 Hospital, Tongji Medical College, Huazhong University of Science and Technology, Wuhan 430022, China; ^3^Department of Neurology, Puai Hospital, Tongji Medical College, Huazhong University of Science and Technology, Wuhan 430033, China; ^4^Department of Infectious Diseases, Union Hospital, Tongji Medical College, Huazhong University of Science and Technology, Wuhan 430022, China

## Abstract

Atherosclerosis is a chronic progressive inflammation course, which could induce life-threatening diseases such as stroke and myocardial infarction. Optimal medical treatments for atherosclerotic risk factors with current antihypertensive and lipid-lowering drugs (for example, statins) are widely used in clinical practice. However, many patients with established disease still continue to have recurrent cardiovascular events in spite of treatment with a state-of-the-art therapy. Atherosclerotic cardiovascular disease (ASCVD) remains the leading cause of mortality worldwide. Hence, current treatment of atherosclerosis is still far from being satisfactory. Recently, M2 macrophages have been found associated with atherosclerosis regression. The M2 phenotype can secrete anti-inflammatory factors such as IL-10 and TGF-*β*, promote tissue remodeling and repairing through collagen formation, and clear dying cells and debris by efferocytosis. Therefore, modulators targeting macrophages' polarization to the M2 phenotype could be another promising treatment strategy for atherosclerosis. Two main signaling pathways, the Akt/mTORC/LXR pathway and the JAK/STAT6 pathway, are found playing important roles in M2 polarization. In addition, researchers have reported several potential approaches to modulate M2 polarization. Inhibiting or activating some kinds of enzymes, affecting transcription factors, or acting on several membrane receptors could regulate the polarization of the M2 phenotype. Besides, biomolecules, for example vitamin D, were found to affect the process of M2 polarization. Pomegranate juice could promote M2 polarization via unclear mechanism. In this review, we will discuss how M2 macrophages affect atherosclerosis regression, signal transduction in M2 polarization, and outline potential targets and compounds that affect M2 polarization, thus controlling the progress of atherosclerosis.

## 1. Introduction

Atherosclerotic cardiovascular disease (ASCVD), including coronary heart disease (CHD), stroke, and peripheral arterial disease, all of the presumed atherosclerotic origin, is the leading cause of mortality worldwide [1]. On the basis of NHANES data, the prevalence of cardiovascular disease (CVD) in adults ≥ 20 years of age increases with age in both males and females. In 2015, 2712630 resident deaths were registered in the United States and CVD was the most important cause of 10 leading causes of deaths. Coronary heart disease (CHD) (43.8%) is the leading cause of deaths attributable to CVD in the United States, followed by stroke (16.8%), high blood pressure (BP) (9.4%), heart failure (HF) (9.0%), diseases of the arteries (3.1%), and other CVDs (17.9%) [2]. Stroke is an acute cerebrovascular disease which brings about severe death threat and residual symptoms, along with heavy life burden. In East Asia, stroke ranks as the first of the top five causes of years of life lost (YLLs), followed by ischemic heart disease, road injury, chronic obstructive pulmonary disorder, and lung cancer [3]. On average, every 40 seconds, one person dies of stroke in the United States [4]. Stroke deaths took up 11.8% of total deaths worldwide, making it the second leading cause of global death in 2013 [3]. Atherosclerosis, including large artery atherosclerosis and intracranial atherosclerosis, is recognized as a major risk factor of stroke [5]. Actually, atherosclerosis is the underlying cause of most clinical cardiovascular events [4], including heart attacks which could lead to acute death.

Researchers have revealed that atherosclerosis is a chronic progressive course. It mainly starts by apolipoprotein B-containing lipoproteins' (Apo-LPs) accumulation in the blood vessel intima, preceded by foam cell formation, and ends up with atherosclerosis plaque shaping as well as artery stenosis [6]. LDL-lowering therapies especially statins, antioxidant therapy, and other classical therapies are widely used for atherosclerosis regression. However, the treatment's efficacy is not satisfactory. A proportion of patients are prevented from continuing long-term use of statins for their adverse effects including myopathy, new-onset diabetes mellitus, and hemorrhagic stroke, and the abrupt cessation of statin treatment can be devastating for heart attacks or strokes [7]. Besides, many patients with established disease still continue to have recurrent cardiovascular events in spite of treatment with a state-of-the-art therapy. In 2015, approximately 17.9 million deaths were attributed to cardiovascular disease globally, which amounted to an increase of 12.5% compared with those in 2005 [8]. Atherosclerotic cardiovascular disease (ASCVD) remains the leading cause of mortality worldwide. Therefore, it is necessary for researchers and clinical physicians to explore other approaches or medicines for antiatherosclerosis therapy.

Recently, M2 macrophage, also termed as the alternatively activated macrophage, has been reported to be playing an important role in the regression of atherosclerotic plaque [6, 9]. Researches show that the M2 phenotype could secrete anti-inflammatory factors [10], promote tissue remodeling and repair [11, 12], and clear dying cells and debris [13], thus contributing to atheroma therapy. Therefore, promoting macrophages' polarization to the M2 phenotype would be a promising strategy for atherosclerosis treatment. Besides, several compounds and medicines are found moderating M2 polarization. Two main signaling pathways of M2 polarization have been studied and bring about several targets for macrophage skewing [14].

In this review, we will discuss the function of M2 macrophages on atherosclerosis regression, signal pathways of M2 polarization, and outline potential targets and compounds that affect M2 polarization.

## 2. Biological Characteristics of M2 Macrophages

### 2.1. Origin of M2 Macrophages

#### 2.1.1. Origin of the Concept of Macrophage Classification

Macrophages, generated by monocytes [15], can be mainly classified into two phenotypes, comprising classically activated macrophage (M1 macrophage) and alternatively activated macrophage (M2 macrophage). The concept of macrophage classification raised in the 1960s, when the term “classical activation” was first used [16]. Decades later, alternatively activated macrophages were identified in the late 1980s in human blood monocyte-derived macrophages and mouse peritoneal macrophages in in vitro experimental settings [17]. Besides, the names of M1 and M2 were decided because M1 and M2 macrophages promoted T1 and T2 responses, respectively [18]. Atherosclerosis was associated with M1- or M2-type responses. Actually, in the early stage, the phenotype classification was extensively described mainly in vitro. Soon after, researches showed that the microenvironment, such as some cytokines, lipids, iron, and calcium, played a critical role in macrophage differentiation both in vitro and in vivo [19, 20]. Macrophages existing in various locations of atherosclerosis lesions are sensitive to their complex microenvironment [21], which could promote macrophage activation and polarization in particular states.

#### 2.1.2. Origin of M2 Macrophages and Monocytes in Atherosclerosis

Macrophages contributing to atherosclerosis can be derived from either monocyte precursors, which infiltrate into the intima and differentiate in response to their microenvironment, or tissue residual macrophages which were embryonically seeded in tissues and are maintained through self-renewal [22]. There are two major subsets of monocytes in murine: Ly6C^hi^ and Ly6C^lo^ monocytes, corresponding to human CD14^hi^ CD16– and CD14+CD16+ monocytes, respectively. The Ly6C^hi^ monocytes employ both CCR2 and CX3CR1 to enter atherosclerotic lesions and are thought to become M1 macrophages in most inflammatory sites. The Ly6C^lo^ monocytes patrol blood vessels and can accumulate partially by CCR5 at inflammatory sites, where they are thought to give rise to M2 macrophages. In atherosclerosis progression, both major subsets of monocytes enter atherosclerotic plaques and Ly6C^hi^ monocytes take a great part of the monocyte phenotypes [23]. In the aorta of Western diet-fed ApoE^−/−^ mice, more of Ly6C^hi^ cells than Ly6C^lo^ cells are found and they are reported to possess higher capacity to form foam cells compared to Ly6C^lo^ monocytes for their higher CD36 expression [24]. Another research shows that in atherosclerosis regression animal models, deficiency of CCR2 or CX3CR1, but not CCR5, prevents plaque regression and acute inhibition of CCR2 prevents plaque regression and suppresses enrichment in the M2 macrophage phenotype [25]. In the model, an ApoE-deficient (ApoE^–/–^) C57BL/6 mouse aortic arch segment with atherosclerotic plaques is transplanted into the abdominal aorta of a WT C57BL/6 recipient mouse. These results infer that atherosclerosis regression after lipid lowering is dependent on the recruitment of Ly6C^hi^ monocytes and their polarization towards the M2 phenotype. The M2 macrophages in atherosclerosis regression are overwhelmingly derived from the newly recruited Ly6C^hi^ monocytes. Besides, the Ly6C^lo^ monocyte population is not required for the promotion of atherosclerosis regression. We suppose that there may be a state of M1-like macrophages after Ly6C^hi^ monocytes polarizing to the M2 macrophage during atherosclerosis progression. It also infers that M2 macrophage polarization contributes to the reduction of atherosclerotic plaques. It seems to be a novel treatment for atheroma to promote M2 macrophage polarization.

### 2.2. Identification of M2 Macrophages

Usually stimulated by interleukin-4 (IL-4) and interleukin-13 (IL-13) in vitro, M2 macrophages express numerous surface markers and produce cytokines and chemokines (Table 1). Reports showed that the mannose receptor (MR/CD206), MHCII, IL-12^low^, CD86, and CD163 were detected on the cell membrane of M2 macrophages and tumor necrosis factor *α* (TNF*α*^low^) as well as the vascular endothelial growth factor (VEGF) also characterized the M2 phenotype [19, 26–28]. Class A scavenger receptor (SR-A) is a pattern recognition receptor primarily expressed in M2 macrophages [29]. As a type of immune cell involved in innate immunity, M2 macrophages can produce proinflammatory cytokines including IL-10 and transforming growth factor beta (TGF-*β*) [30]. Besides, metalloproteinase 12 (MMP12), MMP7, chemokine (C-C motif) ligand 17 (CCL17), CCL22, CCL18, oxidized low-density lipoprotein (PTX3), and IL-R*α* have all been confirmed as the products of M2 macrophages [31, 32]. They also express Mer receptor kinase (MERTK) which plays a key role in efferocytosis [13]. Arginase-1 (Arg1) is a hallmark of M2 macrophages [33–35]. In addition, Arg1, found in the inflammatory zone 1 (Fizz1), and the association of the chitinase 3-like 3 lectin (also referred as Ym1) are commonly used as M2 signature genes in experiments when the M2 macrophage is initiated by IL-4 [10, 14, 36]. Furthermore, Mrc1 mRNA, as another cellular marker of M2 macrophages, is induced comparably by IL-4 and IL-13 [10]. In fact, the M2 program is accompanied with upregulation of C-type lectins, mannose receptor, chitinase family proteins, resistin-like molecules, and IL-10. However, differences and heterogeneity exist in macrophages in human beings and murine. Further researches regarding structure characteristics, cell markers, and functions of M2 macrophages in humans are needed.

## 3. M2 Macrophages and Atherosclerosis

### 3.1. Macrophages in Atherosclerosis Progression and Regression

#### 3.1.1. The Formation Process of Atherosclerosis Plaque

Atherosclerosis is a chronic inflammation disease in which macrophages are the central players in the development and progression of atherosclerotic plaque [37, 38]. Endothelial dysfunction underlies atherogenesis. In fact, all the risk factors of atherosclerosis, including elevated and modified LDL; free radicals caused by cigarette smoking, hypertension, and diabetes mellitus; genetic alterations; elevated plasma homocysteine concentrations; and infectious microorganisms, could cause endothelial damage to some degree [39]. Then, apolipoprotein B-containing lipoproteins (Apo-LPs) accumulate in the blood vessel intima where damage exists. Besides, the injury could induce inflammation, resulting in increased numbers of monocyte-derived macrophages and lymphocytes, which both emigrate from the blood and multiply within the lesion. Via macropinocytosis, phagocytosis of aggregated LDL, and scavenger receptor-mediated uptake (including by scavenger receptor A1 (SR-A1), lectin-like oxidized LDL receptor 1 (LOX1), SR-B1, and CD36), macrophages internalize native low-density lipoprotein (LDL) and very low-density lipoprotein (VLDL) as well as oxidized lipoproteins in the plaque, leading to foam cell formation [40]. Combined with migration and proliferation of smooth muscle cells, cytokines, chemokines, and growth factor that the lesion produced, foam cells constitute the primary part of fatty streaks which then proceed with atherosclerosis plaque genesis.

#### 3.1.2. Metabolic Changes of Macrophages during Atherosclerotic Plaque Progression

**Figure 1:**
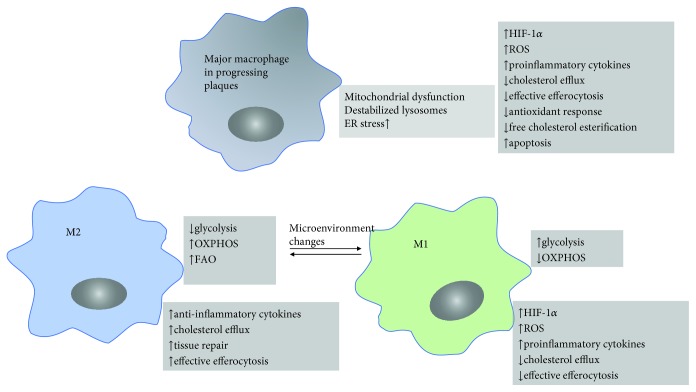
Metabolic features and physical characteristics of M1 macrophages, M2 macrophages, and the major macrophage in progressing atherosclerotic plaques.

Microenvironmental features such as a variety of proinflammatory cytokines, oxidized lipids, cholesterol crystals, oxidative stress, and danger-associated molecular patterns derived from dying cells drive changes in macrophage function alongside metabolic adaptations and consequently facilitate cellular effector responses in atherosclerosis. In vitro and in vivo, the metabolic and effector responses are found different between M1 and M2 macrophages. M1 macrophages induced by LPS show increased glucose uptake, impaired oxidative phosphorylation (OXPHOS) via the tricarboxylic acid (TCA) cycle, abundant reactive oxygen species (ROS) release, and inflammatory cytokine production as Figure 1 illustrated. They also exhibit elevated lipid accumulation, which together contribute to the acceleration of atherosclerosis [41]. On the contrary, evidence shows that M2 macrophages locate far from the lipid core of the plaque and contain smaller lipid droplets compared to M1 macrophages based on a histological analysis [42]. Besides, M2 macrophages highly express opsonins and receptors involved in phagocytosis, resulting in high phagocytic activity. Taken together, these results suggest that M2 macrophages display low cholesterol handling but high phagocytosis. M2 macrophages stimulated by IL-4 exhibit a precipitous increase in fatty acid oxidation (FAO) and OXPHOS which provides the main source of ATP and thereby maintains the cholesterol efflux and efferocytosis capacity of the M2 macrophage. The increased FAO and OXPHOS are also thought to contribute to anti-inflammatory responses [43]. As for macrophages in progressing atherosclerotic plaques, mitochondrial dysfunction, destabilized lysosomes, and high ER (endoplasmic reticulum) stress are reported and result in lower ATP production, decreased cholesterol efflux, increased ROS and proinflammatory cytokine release, and defective efferocytosis. All these results, taken together, suggest that M1 macrophages as the major phenotype of macrophages during atherosclerosis progression and M1 polarization exist. Therefore, microenvironment changes which shift M1 macrophages towards M2 macrophages may change the metabolic style as well as physiology function of M1 into an M2 style, beneficial to reverse atherosclerosis.

#### 3.1.3. M1 and M2 Macrophages in Atherosclerotic Plaque

Recently, researches show that macrophages in atherosclerosis lesions can divide into two main phenotypes: M1 macrophages and M2 macrophages [44, 45]. According to one study of macrophage phenotypes during the progression of atherosclerosis in *ApoE*^–/–^ mice, serial immunohistological examinations showed that plaque macrophages have M2 phenotypes at the early stages of the disease but become M1 macrophages as the lesions advance [34]. A study based on atherosclerosis regression animal models found that atherosclerosis regression after lipid lowering is dependent on the recruitment of Ly6C^hi^ monocytes and their polarization towards the M2 phenotype [25]. These results may infer that the balance between the M1 macrophage and M2 macrophage in plaques is associated with the formation and regression of atherosclerosis. M2 macrophages appearing at the initiation of atherosclerosis may be affected by the microenvironment which leads to M1 polarization and the followed atherosclerotic plaque progression. When the microenvironment changes, such as altered cholesterol metabolism or oxidative stress, the M1 macrophages may therefore retain plasticity and reclaim characteristics of the original M2 phenotype which induce atherosclerosis regression.

**Figure 2:**
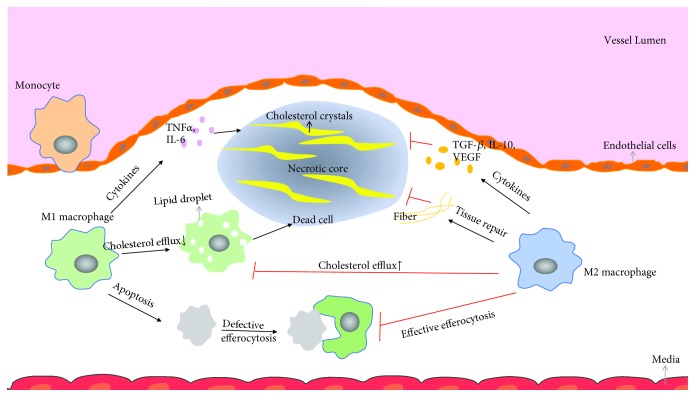
How M2 macrophages affect atherosclerosis development.

In addition, investigators have implicated that M2 macrophages could solve plaque inflammation for its function of secreting anti-inflammation cytokines, promoting tissue repair and effective efferocytosis [6], which is contrary to M1 macrophages and is beneficial to atherosclerosis regression as Figure 2 described. However, much of the theory in this area has been driven by *in vitro* studies and there are important differences in the characterization and roles of macrophage subsets in humans vs. mice.

### 3.2. M2 Macrophage Secretes Anti-Inflammatory Cytokines

M2, as a type of immune cells, can secrete anti-inflammatory cytokines. Recent data confirms that M2 macrophages can produce IL-4 and IL-13 which could induce M2 polarization in turn [46]. IL-10 is an important product of M2 macrophages. IL-10 can affect the morphology of IL-4 and IL-13 on macrophages (for example, rounding versus fusion), can downregulate the expression of MHC class II molecules, and has variable influences on mannose receptor expression, leading to decreased fluid-phase and mannose receptor-mediated endocytosis [20].TGF-*β* is another hallmark secretion of M2 macrophages. Vodovotz and colleagues [47] posed that TGF-*β* could uniquely inhibit inflammation through reducing iNOS-specific activity and decreasing iNOS protein production. Moreover, other cytokines including arginase, EGF, VEGF, IL-6, TNF, and IL-1 were observed in M2-type responses (Table 1) [48].

Moore et al. have put forward that polarizing macrophage to an M2 phenotype would be one approach to reduce the inflammatory state of plaque macrophages and be particularly important in regressing atherosclerosis plaque [6]. Besides, Cardilo-Reis et al. supported that IL-13 could promote atherosclerosis regression through inducing M2 polarization [46]. In their experiment, LDLR^−/−^ mice were divided randomly into two groups and injected intraperitoneally with PBS or IL-13 twice per week. More M2 macrophages were found in lesions of LDLR^−/−^ mice injected with IL-13 than those injected with PBS in vitro, and increased clearance of ox-LDL was detected in M2-abundant atherosclerotic lesion in vitro. Consistently, Khallou-Laschet and his colleagues found that regardless of the atherosclerosis stage, the prevalence of the M2 phenotype was associated with smaller plaque surface areas in ApoE KO mice [34, 49]. Besides, M2 macrophages were less in the symptomatic plaques than the asymptomatic plaques. As atherosclerosis progressed, M2 macrophages would be suppressed while M1 macrophages became predominant features of the atherosclerotic lesion [50]. Above all, promoting M2 polarization may be a potential treatment for atherosclerosis.

### 3.3. M2 Macrophage Promotes Tissue Repair

Evidence shows that M2 macrophages promote tissue repair in several aspects. The cytokines that M2 macrophages secrete, such as IL-6, TGF-*β*, and IL-10, were reported to be contributing to healing, fibroblast proliferation, and collagen production [31]. Ornithine, EGF, VEGF, and other growth factors that are necessary for repair are reported to be produced by M2 macrophages when “Danger” signals or pathogens present [18]. Besides, mainly affected by secretions of TNF*α* and IFN-*γ*, M2 macrophages develop the function of angiogenesis [26], a critical step in the wound healing process [51]. M2 macrophages could synthesize certain repairing-related components, including collagen type VI, fibronectin, and *β*IG-H3 [52, 53]. *β*IG-H3 promoted adhesion and migration of monocytes and fibroblasts, thereby increasing fibroblast collagen production [54]. In addition, when damage occurred, tissue-resident M2 macrophages could participate in the repairing directly [11, 55]. In the cervical region of maternal, increased presence of M2 macrophages was observed during labor and immediately after birth, indicating a tissue-repairing function of M2 macrophages [56]. Accordingly, we propose that M2 macrophages may play a part in atherosclerosis regression.

### 3.4. Effective Efferocytosis of M2 Macrophages

Endocytosis is a basic function of macrophages. In advanced atheroma, defective efferocytosis, representing inefficient clearance of dying cells, debris, and apoptotic macrophages by phagocytes, could bring about atherosclerotic plaque necrosis, thus inducing acute cardiovascular and cerebrovascular events. It was noted that M2 macrophages expressed high levels of MERTK (also known as Eyk, Nyk, and Tyro-12), a tyrosine kinase receptor which bridged apoptotic cells to phagocytes, thereby increasing the efferocytosis of dying macrophages [13]. Thorp and his colleagues [57] also supported that MERTK could suppress macrophage inflammation and work in the same direction as LRP-1 which promoted macrophage survival. In addition, the M1 phenotype displays a delay in phagosome fusion with lysosomes and then plays a negative role in efferocytosis [58]. Therefore, skewing macrophages to an M2 phenotype may be beneficial to efficient efferocytosis, stabilizing atherosclerotic plaque, and may be conducive to atherosclerosis regression.

However, Stienstra et al. showed that M2 macrophages were more susceptible for foam cell formation than M1 macrophages for their rapidly accumulating oxidized LDL [9]. One other study approved that silencing the leukocyte-associated immunoglobulin-like receptor 1 (LAIR-1) in macrophages could promote M2 macrophage polarization and increase foam cell formation [59]. Both the above two studies test the intracellular lipid accumulation by Oil Red O staining after macrophages are being exposed to oxidized low-density lipoprotein (ox-LDL). The former found that M2 macrophages showed intense Oil Red O staining after exposure to oxidized LDL for 24 or 48 hours. In the latter study, macrophages were serum starved for 8 hours and then exposed to ox-LDL or LAIR-1 siRNA or a combined treatment of both for 4 hours. The Oil Red O staining results showed that the LAIR-1 siRNA group displayed a marked lipid storage capacity compared to the control group and the combined treatment caused a dramatic increase in the accumulation of lipids in THP-1 macrophages. Silencing LAIR-1 was further found to be promoting M2 polarization, evidenced by the fact that the levels of M2 markers such as Arg1 and CD206 were enhanced after silencing LAIR-1. Both the two studies infer that M2 macrophages are more likely to develop into foam cells. However, the studies were only done in vitro and in a cellular level. The functions, including efferocytosis, cytokines production, and cholesterol efflux, of foam cells produced by macrophages in the two studies, were not well studied. If we extend the exposure time to ox-LDL or alter the concentration of ox-LDL, would the level of cholesterol accumulation in macrophages change? Distinct metabolic programs are required to support energy demands of M1 and M2 macrophages. It is not referred whether the cell culture medium provides enough energy to maintain common M2 metabolism or not. The microenvironments in vivo were complicated and quite differ with the in vitro circumstance. Whether they could accelerate atherosclerosis formation in vivo remains to be studied. Actually, evidence suggests that it is M1-derived foam cells that could induce endothelial-mesenchymal transition (EndMT) by upregulating CCL4 and increase endothelial permeability and monocyte adhesion which is subsequently followed with plaque formation [60]. Rahman and his colleagues demonstrated that M2 polarization plays an essential role in atherosclerosis regression in a mouse model. Accordingly, M2 polarization promotion would still be a promising strategy for atherosclerosis treatment. Noticeably, the function of the M2 macrophage, the cell morphology, and the physical changes alongside atherosclerosis development are worth to be further studied.

## 4. Signaling Pathways of M2 Polarization

### 4.1. The JAK-STAT Pathway

**Figure 3:**
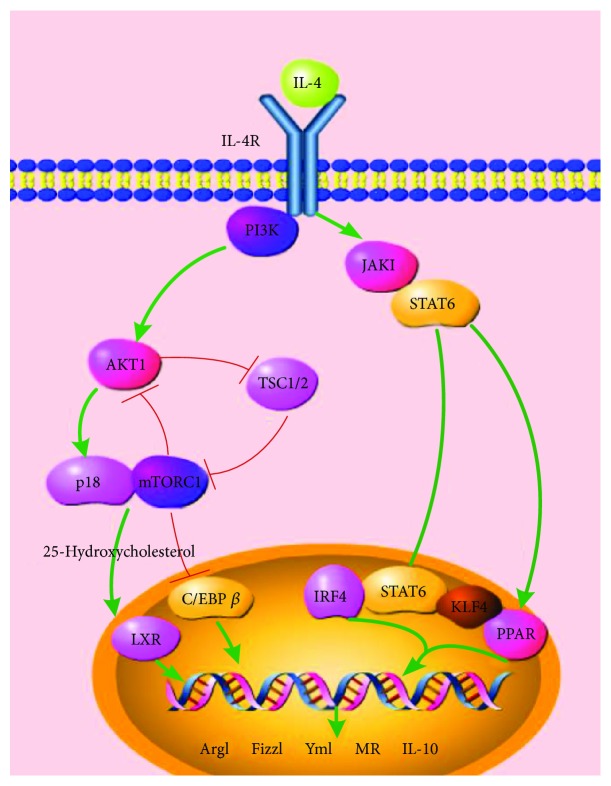
M2 polarization pathways. The JAK-STAT pathway and Akt-p18-mTOR-LXR pathway are recognized as the main two pathways leading to M2 macrophage polarization. TSC1/2 was involved in the Akt-p18-mTOR-LXR pathway and affects macrophage polarity.

The JAK-STAT pathway has already been recognized as a classical pathway leading to M2 polarization in vitro and in vivo. JAK1, a member of the Janus kinase family, has been reported to associate with components of the IL-4R complex [61, 62]. Initiated by IL-4 or IL-13 combining with the receptors on the cell membrane, JAK1 will be phosphorylated and then will activate STAT6, leading to the upregulation of M2-like genes, including Ym1, Arg1, Fizz1, IL-10, and MGL1 [10, 61, 63] (Figure 3).

The nuclear receptor peroxisome proliferator-activated receptor-*γ* (PPAR*γ*) plays a key role in regulating lipid metabolism and inhibiting proinflammatory gene expression in macrophages [64]. Recent evidences supported that PPAR*γ* deficit could negatively affect M2 polarization and that the STAT6 transcription factor was a facilitator of PPAR*γ*-regulated gene expression in macrophages [65], suggesting a crosstalk between PPAR*γ* and the JAK–STAT6 axis in M2 polarization [66]. Besides, researches showed that PPAR*δ* could be activated by pSTAT6 and thereby inhibited JNK, causing the inhibition of M1 macrophages and upregulation of M2 macrophages [63]. Recently, IRF4 was reported to polarize macrophages to an M2 phenotype in response to parasites or the fungal cell wall component chitin [67] and it was posed that IRF4 may take part in the JAK-STAT axis or the Akt-mTOR signal in M2 polarization [66]. The Krüppel-like factor (KLF) family members are thought to play important roles in cellular differentiation. Results demonstrate that KLF4 cooperates with STAT6 in promoting M2 macrophage polarization [68, 69]. Deletion of the KLF4 gene in macrophages disrupts M2 function and increases proinflammatory gene expression [68]. The fact that PPAR*γ* levels are reduced in KLF4-null cells and tissues raises the possibility that KLF4 and STAT6 may also cooperate to augment PPAR*γ* expression [70]. It is as well supported by the result that myeloid deletion of PPAR*γ* leads to a phenotype that is similar to that of KLF4 Mye-KO mice (myeloid-specific KLF4-deficient mice). On the other side, both in vivo and in vitro experiments find that KLF4 could inhibit M1 polarization by inhibiting NF-*κ*B activation [71]. Thus, KLF4 may play a crucial role in M2 polarization.

### 4.2. The Akt-p18-mTOR-LXR Pathway

When macrophages are treated with IL-4, Akt will be activated by PI3K. Thereafter, Lamtor1 together with amino acid-activated mTORC1, with 25-hydroxycholestero existing, could initiate LXR which will promote M2 gene expression [14, 72]. Akt protein kinase, a member of AGC kinases (AMP/GMP kinase and PKC subfamily of proteins), plays important roles in many cellular functions including proliferation, migration, cell growth, and metabolism [73]. Two main isoforms of Akt kinase, Akt1 and Akt2, take part in and contribute differentially to macrophage polarization. In vitro and in vivo, studies observed that knockdown of Akt2 increased the expression of Arg1, Fizz1, and Ym1 in macrophages while Akt1 ablation promoted high levels of iNOS expression and production of NO, TNF*α*, and IL-6 [74]. The results indicate that knockdown of Akt2 promotes M2 polarization and Akt1 ablation contributes to M1 polarization. Otherwise, suppression of Akt2 and activated Akt1 was found associated with increased mitophagy and resistance to apoptosis. To investigated the roles of Akt1 and Akt2 in atherosclerosis, studies generated mice with hematopoietic deficiency of Akt1 or Akt2. After 8 weeks on Western diet, LDLR^−/−^ mice reconstituted with Akt1^−/−^ fetal liver cells showed similar atherosclerotic lesion areas compared with control mice transplanted with wild-type (WT) cells. In contrast, LDLR^−/−^ mice reconstituted with Akt2^−/−^ fetal liver cells had dramatically reduced atherosclerotic lesions compared with control mice transplanted with WT cells in both genders. Peritoneal macrophages isolated from Akt2^−/−^ mice were shifted towards an M2 phenotype and showed decreased expression of proinflammatory genes. These results demonstrate that loss of Akt2 promotes the ability of macrophages to undergo M2 polarization and reduces atherosclerosis progression. Consistently, Rotllan and his colleagues found the same results and provided more evidence that Akt2 regulated cholesterol metabolism and targeting Akt2 in macrophages might be beneficial for atherosclerosis treatment [75].

Lamtor1 (also known as p18) is a type of lysosomal adaptor protein complex regulator and is attached to the lysosome membrane via covalently bound fatty acids, forming a nutrient-sensing complex with lysosomal vacuolar-type H^+^-ATPase (v-ATPase) [76]. Emerging studies have described that Lamtor1 has a critical role in M2 polarization signaling [14]. A study established Lamtor1-deficient bone marrow-derived macrophages (BMDMs), and markedly defective M2 polarization was found in those cells as classic M2 signature genes such as Arg1, MR, and IL-10 were hardly detected. What is more, M1 polarization was enhanced in Lamtor1-deficient BMDMs. Retroviral transfer of the Lamtor1 gene into Lamtor1-deficient BMDMs reversed the M2 signature gene expression [14]. In vivo, myeloid-specific Lamtor1 conditional KO mice exhibited defective polarization of M2 macrophages and reduced IL-10 production, indicating a significant role of Lamtor1 in M2 polarization. Lamtor1 is the scaffold for mTORC1 activation. Lamtor1-deficient BMDMs showed reduced mTORC1 activity and smaller cell sizes than wild-type BMDMs. Full activation of mTORC1 by IL-4 was also dampened by the absence of amino acids. In contrast, activation of mTORC1 by leucine was impaired in Lamtor1-deficient macrophages, confirming the involvement of Lamtor1 in amino acid sensing. Real-time PCR found decreased LXR target genes such as ABCA1 and LPL in Lamtor1-deficient BMDMs as well as in mTOR-inhibited wild-type BMDMs stimulated by IL-4, suggesting that LXR is the downstream transcription factor of Lamtor1 and mTORC1. In Lamtor1-deficient BMDMs, 25-hydroxycholesterol was detected by mass spectrometry and its amount was much smaller than that in wild-type BMDMs before or after macrophage activation. However, the amounts of LXR protein in M0-state wild-type and Lamtor1-deficient BMDMs were comparable and mRNA levels of LXRs in M2-state Lamtor1-deficient BMDMs were not less than that in wild-type counterparts, indicating that Lamtor1 and mTORC1 is required for production of 25-hydroxycholesterol and subsequent activation of LXR in macrophages.

Moreover, target of rapamycin complex 1 (TSC1) is also involved in mTORC1 regulation as Figure 3 illustrated [36, 77]. After IL-4 stimulation, TSC1-deficient peritoneal macrophages as well as TSC1KO bone marrow cells showed decreased M2 polarization as reduced hallmarks including Arg1, Fizz1, and Ym1 were observed. However, blocking mTOR activity by its specific inhibitor Rapa almost completely rescued the M2 polarization deficiency of TSC1 KO macrophages as indicated by the increased hallmark expression. Besides, macrophages derived from mTOR KO bone marrow cells by M-CSF expressed significantly higher levels of Arg1, Fizz1, and Ym1 than WT macrophages after IL-4 stimulation. These studies confirm that enhanced mTOR activity is associated with poor M2 polarization of TSC1KO macrophages. TSC1KO macrophages also presented reduced p-Akt activity and nuclear C/EBP*β* expression after IL-4 treatment. Deletion of mTOR significantly reversed the decreased C/EBP*β* while overexpression of C/EBP*β* rescued the decreased Arg1, Fizz1, and Ym1 expression in TSC1KO macrophages. These results suggest that decreased C/EBP*β* expression in TSC1KO macrophages is induced by overactivated mTOR activity and is related to less M2 polarization [36]. In addition, CREB- (cAMP-responsive element-binding protein-) mediated induction of C/EBP*β* expression was found required for M2-specific gene expression [36, 78]. Lawrence and Natoli also confirmed the CREB–C/EBP axis in the M2 polarization signal [66].

### 4.3. Other Potential Pathways

Except for the above two common signaling pathways, other potential pathways leading to M2 polarization are also studied. Ma et al. approved that ABCA1 in macrophages could promote IL-10 but lessen proinflammatory cytokine secretion [79]. Their studies presented evidence that ABCA1 activated PKA, which would elevate PKA activity and contribute to M2-like inflammatory response. Consistently, cholesterol lowering by statins, methylcyclodextrin, or filipin could also activate PKA and consequently transform macrophages towards the M2 phenotype. Recently, ERK5, a member of the mitogen-activated protein kinase family and highly expressed in monocytes/macrophages, is reported to be related with efferocytosis as well as M2 macrophage polarization. BMDMs isolated from ERK5-MKO mice (ERK5*fl/fl* mice crossed with LysM*Cre^+/−^* (C57BL/6 background) mice) showed higher expression levels of M1-related genes and lower levels of M2-related genes such as Arg1, Fizz1, and YM1 [80]. The atherosclerotic lesion area observed in the en face sample of the aorta was significantly larger in ERK5-MKO mice than in NLC (nontransgenic littermate control) mice. In addition, statins are found that could increase ERK5 kinase activity in macrophages and increase their phagocytic capacity. These results suggest that ERK5 may be a target for M2 polarization and atherosclerosis regression and the effective efferocytosis of the M2 macrophage may partly lie in ERK5 activation in return.

ITC4H, an E3 ubiquitin ligase [81], was reported to modulate macrophage polarization both in vivo and in vitro [82]. Stöhr et al. found that ApoE^−/−^ITCH^−/−^ mice fed a Western diet for 12 weeks showed increased circulating M2 macrophages together with a reduction in plaque formation [83]. Besides, the loss of ITCH reduced atherosclerotic development by preventing the clearance of SREBP2 and thus upregulating the LDL receptor-mediated reuptake of LDL into the liver. We consider that the M2 polarization in ITCH-deficient mice may attribute to the downregulated LDL in the evidence that exposure to oxidized LDL renders M2 macrophage proinflammatory properties [9]. However, inhibiting ITCH may be beneficial to M2 polarization and promote atherosclerosis regression. Some miRNAs including miR-155, miR-124, miR-33, and miRNA let-7c were reported to contribute to M2 gene expression, suggesting a role for miRNA in M2 polarization [84–87].

Accumulating evidence supports a role for endoplasmic reticulum (ER) stress in all stages of the developing atherosclerotic lesion. Researches have shown that ER stress signaling through glycogen synthase kinase-3*α* (GSK-3*α*) may significantly contribute to macrophage lipid accumulation, inflammatory cytokine production, and M1 macrophage polarization [88]. Inhibiting GSK-3*α* might attenuate atherosclerosis and promote M2 polarization. However, there are no currently known specific GSK3*α*/*β* inhibitors. IRE1, the ER-resident transmembrane protein kinase and endoribonuclease, is the most conserved ER stress sensor and was reported to be a critical switch governing M1–M2 macrophage polarization [89]. Myeloid-specific IRE1*α*-knockout mice were created and showed lower body fat mass and increased energy expenditure compared to their counterparts when they were fed a normal chow. In BMDMs derived from myeloid-specific IRE1*α*-knockout mice, IL-4 stimulation of Ym-1 protein, as well as signature M2 marker genes, was significantly elevated but there were no changes in phosphorylation of the transcription factor STAT6. These results demonstrated that loss of IRE1*α* may promote IL-4 induction of M2 polarization in a way different from that of the common M2 polarization pathways. Moreover, there are important differences in the characterization of macrophages in adipose tissue vs. atherosclerotic plaques. Whether IRE1*α* deletion in macrophages would promote M2 polarization and subsequent atherosclerosis regression remains to be further studied.

## 5. Potential Targets and Compound That Promote M2 Polarization

### 5.1. Enzymes as Potential Targets for M2 Polarization

Inhibitors of dipeptidyl peptidase (DPP), such as Gliptins and Sitagliptin, can improve the control of blood glucose levels, enhance insulin sensitivity, and are widely used for the treatment of type 2 diabetic patients (Table 2) [90]. Besides, DPP-4 inhibitors were found reducing blood cholesterol levels and could downregulate the formation of atherosclerosis in both diabetic animal models and nondiabetic conditions [91]. Brenner et al. confirmed that Sitagliptin could promote M2 polarization during monocyte differentiation via the SDF-1/CXCR4 signaling, thus inhibiting the initiation of atherosclerosis [91]. Besides, dipeptidyl peptidase I, also known as cathepsin C (CatC), was reported to upregulate in M1 macrophages, whereas its deficiency led to combined M2 (in vitro) and Th2 polarization (in vivo) [92]. Moreover, studies showed that inhibition of DPP-8/9 activity with compound 1G244 could reduce activation of M1 macrophages for the significantly reduced secretion of the proinflammatory cytokines IL-6 and TNF*α*. However, intriguingly, no M2 marker upregulation was noted [93]. In spite of this, DPP inhibition including DPP-4 inhibition and DPP-8/9 inhibition may be a potential target for M2 polarization and atherosclerosis regression treatment. More researches are needed for DPP in macrophage polarization.

Cao et al. suggested that macrophage histone deacetylase 9 (HDAC9) upregulation was atherogenic through suppressing cholesterol efflux and alternatively activated macrophage skewing in atherosclerosis [94]. In contrast, HDAC9 deficiency in macrophages promoted M2 polarization and decreased M1 inflammatory genes [94]. In fact, HDAC9 is an enzyme that alters chromosome structure and affects transcription factor access to DNA and plays a critical role in transcriptional regulation, cell cycle progression, and developmental events [95]. In their study, HDAC9 deletion led to upregulation of lipid homeostatic genes and downregulation of inflammatory genes and skewed towards an M2 phenotype via increased accumulation of total acetylated H3 and H3K9 by the promoters of ABCA1 (ATP-binding cassette transporter), ABCG1, and PPAR-*γ* (peroxisome proliferator-activated receptor) in macrophages [94]. In addition, evidence showed that protein kinase A (PKA) activated by ABCA1 contributed to transforming macrophages towards an M2-like phenotype [79]. Deficiency or inhibition of HDAC9 and activating PKA are both worth studying to determine their roles on M2 polarization.

Chitinase 1 (CHIT1), secreted by activated macrophages, could curb inflammatory responses, promote lipid uptake and cholesterol efflux in macrophages, and polarize macrophages towards an M2 phenotype, thus exerting protective effects against atherosclerosis [96]. When CHIT1 activity was suppressed using either chitinase inhibitor allosamidin or CHIT1 siRNA transfection, macrophage polarization was affected and skewed towards an M1 phenotype [96]. This indicated that activating CHIT may play a role in promoting M2 skewing.

McAlpine et al. demonstrated that deletion of myeloid glycogen synthase kinase (GSK3*α*) attenuated the progression of atherosclerosis by promoting an M2 macrophage phenotype [97]. Besides, increased P-STAT6 in GSK3*α*-null M2 cells was found, which underscored the role of GSK3*α* activation in shifting macrophages towards an M1 phenotype and promoted atherosclerosis [97]. However, compounds that inhibit GSK3*α* and promote M2 polarization are less and still need to be explored.

Evidence by Aflaki and his colleagues showed that defective lipolysis in macrophages lacking adipose triglyceride lipase (ATGL), the major enzyme responsible for triacylglycerol hydrolysis, favored an anti-inflammatory M2-like macrophage [98]. Their study was carried on adipose triglyceride lipase-deficient (ATGL^−/−^) mice, and the peritoneal macrophages were cultured. Production of proinflammatory IL-6 was decreased in ATGL^−/−^ compared to WT macrophages, whereas the release of the anti-inflammatory cytokines IL-10 and TGF-*β* was upregulated [98]. Anti-inflammation genes and M2 marker genes were also found higher in ATGL^−/−^ macrophages than in WT macrophages. The study indicates that insufficient lipolysis influences macrophage polarization to an M2 phenotype and may impact atheroma development.

In vitro cell experiments revealed that nicotinamide phosphoribosyl transferase (NAMPT) was increased both intracellularly and extracellularly in M1 macrophages compared to M2 macrophages [99]. In addition, inhibiting NAMPT enzymatic activity by FK866 inhibited M1 polarization in macrophages and in contrast enhanced the expression of CD163 and PPAR which were markers of M2 macrophages [99]. However, Audrito et al. have reported that extracellular NAMPT could promote M2 polarization in patients with chronic lymphocytic leukemia (CLL) [100]. Recently, whether extracellular NAMPT could promote M2 skewing in atherosclerosis models and how NAMPT regulates macrophage polarization are not completely understood and more researches on them are expected.

### 5.2. Receptors as Potential Targets for M2 Polarization

Macrophage class A scavenger receptor (SR-A), with multiple endocytic routes in response to various environments, is a multifunctional pattern recognition receptor involved in a range of macrophage-associated pathophysiological processes, including atherosclerosis, diabetes, and myocardial infarction [101–103]. Qian et al. had confirmed that SR-A could moderate macrophage polarization [104]. In myocardial infarction, SR-A attenuated cardiomyocyte necrosis through suppressing M1 macrophage subset polarization, indicating a role for SR-A in steering M2 polarization [105]. Thus, we hypothesize that overexpressing SR-A may be a way to polarize macrophages to an M2 phenotype (Table 3).

Singla et al. reported that when Notch1 receptor (Notch1R) was treated with either the inhibitor DAPT (*γ*-secretase inhibitor or N-[N-(3,5-difluorophenacetyl)-l-alanyl]-S-phenylglycine t-butyl ester) or Notch1R small-interfering RNA (siRNA), M2 marker molecules were raised whereas M1 marker molecules downregulated [106]. They concluded that inhibition of Notch1R and subsequent downstream signaling promoted monocyte to polarize to an M2 macrophage, enhanced anti-inflammatory mediation, and diminished M1 macrophage differentiation [106]. Future researches are warranted. Besides, Notch2R, Notch3R, or Notch4R activation may have the possibility to affect macrophage polarization.

Mallavia et al. proposed that strategies to modulate IgG (immunoglobulin G) Fc*γ* receptors (Fc*γ*R) activating/inhibitory balance and effector functions could suppress atherosclerosis by skewing macrophage inflammatory states into the M2 phenotype [107]. Unanimously, studies indicated that ApoE^−/−^Fc*γ*RIIb^−/−^ double-knockout (DKO) mice induced the presence of M2 macrophages with higher arginase 1 (Arg1) and lower inducible nitric oxide synthase (iNOS) expression than apolipoprotein (ApoE^−/−^) mice [108]. With the absence of Fc*γ*RIIb which existed on B cells and suppresses IgG production, IgG expression raised and produced more IC (IgG antigen immune complexes) which could combine with TLR4 ligands thus producing M2 macrophages [109]. However, this finding was only established on a congenic background. How IC affects TLR4 and whether IC takes part in the above M2 polarization signaling pathway are still under research and worth studying.

Rev-erba (Nr1D1), a member of the nuclear receptor, was reported to increase the appearance of anti-inflammatory M2 macrophages [110]. Some marker molecules of M2 were significantly decreased in macrophages obtained from Rev-erba knockdown mice while overexpression of Rev-erba by Lentivirus-Rev-erba increased the level of M2 macrophages [111]. Besides, a Rev-erba ligand heme which mimicked the overexpression of Nr1D1 promoted M2 marker expression, suggesting that upregulating Nr1D1 may polarize macrophages to an M2 phenotype and that heme is a promising compound for macrophage polarization as well as atherosclerosis treatment.

Syndecan-1 (Sdc-1), a member of cell surface proteoglycans, participates in the regulation of events related to tissue repair and chronic injury responses including cell–substrate interactions, matrix remodeling, and cell migration [112]. Recently, reports showed that Sdc-1 was functionally significant in macrophage polarity in which the Sdc-1 expression on macrophages was associated with anti-inflammatory M2 polarization in both murine and humans [113]. Besides, in animal models, when the intermediate conductance calcium-activated potassium channel, also known as KCa3.1, was blocked by TRAM-34, strikingly, reduction of plaque rupture and luminal thrombus in carotid arteries, decreased repression of M1-related markers, and enhanced expression of M2 markers within the atherosclerotic lesion were observed [114–116]. This suggests that KCa3.1 may be involved in macrophage polarization and its inhibitors may promote M2 skewing.

### 5.3. Transcription Factors as Potential Targets for M2 Polarization

Many studies supported that peroxisome proliferator-activated receptors (PPARs) transcriptionally regulated macrophage activation in obesity, insulin resistance, and cardiovascular disease [117–119]. In mammals, PPARs are classified into 3 subtypes (PPAR*α*, PPAR*δ*, and PPAR *γ*) [120]. Thiazolidinediones (TZDs) such as rosiglitazone and thiazolidinedione are the activators of PPAR*γ* and are used to treat type 2 diabetes. Bouhlel et al. confirmed that M2 marker expression was related to PPAR*γ* in human atherosclerotic lesions [121]. Besides, activating PPAR*γ* with TZDs or the GW1929 compound polarized human monocytes, but not M1-programmed macrophages, foam cells or resting macrophages, to an anti-inflammatory M2 phenotype [121]. Yamamoto et al. showed that combining the PPAR*γ* agonist, pioglitazone, together with an ARB, losartan, could reduce renal injury-initiated progression of atherosclerosis through raising the ratio of M2/M1 phenotype macrophages [122]. Consistently, reports showed that the treatment of GW1516, a PPAR*δ* agonist, upregulated M2 cytokines, while it decreased the expression of M1 cytokines, suggesting a macrophage steering function of PPAR*δ* agonists (Table 4) [123]. Intriguingly, PPAR*α* agonists such as fenofibrate can regulate lipoprotein metabolism but nearly no macrophage skewing effect has been suggested in recent researches.

The neuron-derived orphan receptor 1 (NOR1) working as a transcription factor could steer macrophage polarity in humans. De Paoli et al. supported that NOR1 was a direct target of STAT6 which was involved in M2 polarization and NOR1 silencing by siRNA in an M2 macrophage decreased the expression of M2 markers indicating a critical role of NOR1 in M2 polarity [124]. Accordingly, NOR1 could be a promising pharmacological target for balancing macrophage skewing in atherosclerosis plaques. Besides, Liao et al. indicated that Krüppel-like factor 4 (KLF4) promoted M2 marker expression by cooperating with STAT6 which can induce KLF in turn [70]. Studies also supported that KLF was related to the expression of PPAR*γ*, thus in the other way regulating M2 polarization [70, 125, 126]. An animal experiment demonstrated that Kallistatin (KS) inhibited atherosclerotic plaque formation through promoting M2 polarization via Krüppel-like factor 4 activation. In cultured macrophages, KS significantly stimulated M2 marker expression and decreased M1 marker expression, as determined by flow cytometry and real-time polymerase chain reaction [127]. These effects were blocked by KLF4 small-interfering RNA oligonucleotides. It seems that KLF4 will be a critical target for macrophage skewing and activating KLF4 may benefit atherosclerosis regression.

Tsuchiya found that genetic ablation of forkhead transcription factors (FoxO) could increase atherosclerosis development in low-density lipoprotein receptor knockout mice, indicating an atherosclerosis protective role of FoxO [128]. In addition, decreased Akt phosphorylation was reported in FoxO-deficient mice models [129]. We consider whether FoxO participates in M2 polarization in the evidence that Akt signals in the M2 polarization pathway. Chung et al. provided evidence that in hyperglycemia, FoxO1 was necessary for regulating the macrophage phenotype through increasing IL-10 gene expression [130]. Kawano et al. consistently approved that Pdk1-FoxO1 pathway was required for the activation of alternative macrophages [131]. We thus recognize FoxO1 as a potential target for M2 polarization.

Researchers recently showed that the transducin-like enhancer of split-1 (TLE1) was abundant in alternative macrophages both in vitro and in vivo in human atherosclerotic plaques [132]. Besides, M2 markers including TGF-*β* and IL-10 were observed decreasing when TLE1 was silenced by siRNA in alternative macrophages [132]. This research suggests a new role for TLE1 in macrophage polarization except the common functions including regulating the transcription of genes related to developmental processes, neurogenesis, myogenesis, and cell survival [133]. However, studies committed to this new role of TLE1 are few and more focuses are needed to investigate the mechanism of TLE1 in M2 polarization.

### 5.4. Biomolecules Affecting M2 Polarization

#### 5.4.1. Oxysterol Mixture and Vitamin D

Studies of Marengo and his colleagues showed that oxysterol mixture (OxMix), especially 27-hydroxycholesterol (27-OH), could drive M2 polarization of human macrophages [134]. During their experiment, monocyte cells obtained from peripheral blood mononuclear cells (PBMC) of healthy donors were treated into M0 stage macrophages. Thereafter, OxMix, 27-OH cholesterol, and ethanol (EtOH) were given to the macrophages. The results showed that OxMix as well as 27-OH cholesterol reduced the expression of CD36 and CD204 and declined the reactive oxygen species (ROS) levels which predicted a downregulation of M1 macrophages or a rising of M2 macrophages [135]. In addition, increased secretion of IL-10 and expression of LXR and ABCA1 were found in 27-OH-treated macrophages, thus further confirming an M2 skewing function of OxMix, in particular, 27-hydroxycholesterol. Consistent with the above research, 1,25 (OH)_2_D_3_ was found to polarize macrophages to an M2 phenotype by Yin et al. [136]. They argued that 1,25 (OH)_2_D_3_ induced M2 polarization in macrophage-derived foam cells via increasing 27-OH levels. Besides, they also suggested that 27-OH induced ABCA1 and ABCG1 expression and that the 27-OH/LXR*α* pathway played a crucial role in promoting macrophage cholesterol efflux and anti-inflammation effect. Accordingly, we conclude that 27-hydroxycholesterol, 1,25 (OH)_2_D_3_, and other OxMix may be promising treatments for M2 polarization and atherosclerosis regression.

In addition, vitamin D (VD), which can be activated and transformed into 1,25 (OH)_2_D_3_ in vivo, may also affect the process of M2 polarization. Reports showed that VD reduced endoplasmic reticulum (ER) stress thus slowing down the development of atherosclerosis [137, 138]. Interestingly, the suppression of ER stress by VD was accompanied by upregulated M1 macrophages and stimulated ER stress promoted higher levels of “M2 macrophages” which were beneficial to foam cell formation and atherosclerosis plaque development [139–141]. We contribute this to the difference of experiment models and environment and suppose that ER stress may have an impact on the features of M2 macrophages [9]. More researches about how circumstances affect macrophage polarization and functions are still needed.

#### 5.4.2. Others

*(1) Adiponectin*. Adiponectin is recognized as a critical vasculoprotective protein with insulin-sensitizing and anti-inflammatory functions [142, 143]. Lovren et al. reported that primary human monocytes were differentiated into M2 macrophages in the presence of adiponectin and that macrophages obtained from adiponectin knockout mice showed diminished levels of M2 markers especially MR, which were restored with adiponectin treatment [144]. Their studies concluded that adiponectin promoted human monocytes to polarize into alternative anti-inflammatory M2 macrophages and inhibited the development of atherosclerosis (Table 5). Mandal et al. held the view that full-length adiponectin (flAcrp) potently shifted the polarity of Kupffer cells and RAW264.7 macrophages to an M2 phenotype via the adiponectin R2 receptor [145]. Besides, IL-4/STAT6 signaling was needed in flAcrp induced-M2 polarization from RAW264.7 macrophages. In addition, Fukushima et al. found that decreased adiponectin was related with lower levels of M2 markers such as IL-10 and arginase-1 in the liver of mice, indicating that adiponectin might induce the recruitment of “M2-polarized Kupffer cells” [146]. Ohashi and his colleagues also approved that adiponectin promoted a shift to an anti-inflammatory phenotype macrophage in cultured murine and human macrophages [147]. On the contrary, Cheng et al. argued that adiponectin promoted neither classical (M1) nor alternative (M2) macrophage activation but initiated a proinflammatory response that resembled M1 macrophages more closely than M2 macrophages and many M1 marker genes were tested after treated with adiponectin [148]. Actually, the latter demonstrated that adiponectin could not activate M2 macrophages as well as M2 polarization, while the former indicated that adiponectin promoted monocytes to skew to M2 macrophages. Accordingly, more researches are needed on adiponectin about macrophage polarization because adiponectin seems to be a promising compound for M2 polarization and atherosclerosis regression.

*(2) Semaphorin 3E (Sema3E)*. The Semaphorins are a large family of neuronal guidance cues which affect vascular development and neuroimmune signaling [149–151]. Wanschel et al. demonstrated that M1 macrophages highly expressed Sema3E while M2 macrophages did not and found that the decreased level of Sema3E in regressing atherosclerotic plaques was related to macrophage phenotype shifting from a predominant M1 phenotype to an M2 phenotype [152]. Their research indicated that inhibiting Sema3E might promote M2 polarization and downregulate the inflammation in atherosclerotic lesions. However, studies about this are less and more attentions are worth paying.

*(3) Bone Morphogenetic Protein-7 (BMP-7)*. Singla et al. provided evidence that BMP7 promoted M2 macrophage polarization and anti-inflammation cytokine release and would benefit atherosclerotic plaque reversion [153]. They showed that in vitro BMP-7 upregulated BMP-7R expression which leaded to activate PI3K, Akt, and mTOR, thus enhancing M2 macrophage polarization. In vivo, higher levels of M2 macrophages were found in PLAC models treated with BMP-7 compared to the sham and partial left carotid artery ligation groups (PLCA). All indicate that BMP-7 may play a role in M2 generation or polarization. We wonder if BMP-7 may be used to induce M2 polarization and treat atherosclerosis.

*(4) Thioredoxin (Trx)*. Thioredoxin-1 (Trx-1) is an oxidative stress-limiting protein with anti-inflammatory and antiapoptotic properties. Hadri et al. found that M2 markers including CD206 and IL-10 were elevated in macrophages treated by Trx-1 and exposed to IL-4 or IL-4/IL-13 in vitro while M1 markers such as tumor necrosis factor-*α* and monocyte chemoattractant protein-1 were downregulated [154]. They confirmed that Trx-1 promoted M2 polarization via downregulation of p16^INK4a^. Besides, Trx-1 colocalized with M2 macrophages in human atherosclerotic lesions indicating a potential function of Trx-1 to shift macrophages to an M2 phenotype. However, its truncated form (Trx-80) was reported to initiate inflammation and boost atherosclerotic plaque formation [155]. Accordingly, motivating Trx-1 or inhibiting the produce of Trx-80 may exert an M2 polarization effect and decrease the atheroma lesion area.

*(5) ApoA-I and Myeloperoxidase*. Preclinical and clinical studies have shown that apolipoprotein A-I (ApoA-I) is beneficial to decrease atherosclerosis lesion [156–158]. Hewing et al. showed that native ApoA-I injections could cause a significant increase in anti-inflammatory M2 macrophage markers and decrease in inflammatory M1 macrophages in atherosclerotic plaques [159]. Myeloperoxidase targeted at ApoA-I and could lead to dysfunction of ApoA-I. Inhibiting or extenuating the activity of myeloperoxidase might protect ApoA-I and then increase the polarization of M2 macrophages. Even though ApoA-I is the major protein constituent of high-density lipoprotein (HDL), HDL does not affect the polarization of human monocytes towards an M2 phenotype and studies on the therapeutic effect of HDL are still being conducted [160, 161].

*(6) Nitroxyl Anion (HNO) Donors*. HNO is a one-electron reduced and protonated form of NO^•^. HNO donors have been recognized as an attractive addition to the current treatment for patients with acute heart disease and modulate cardiac function [162]. Recently, HNO was found to promote M2 macrophage polarization as well as reduce endothelial and monocyte activation [163]. The study reported increased CD200R and CD206 expression, mRNA gene expression of CD206, and the anti-inflammatory gene scavenger receptor B1 (SR-B1) in macrophages treated with IL-4 coincubated with Angeli's salt (AS). This indicates that HNO donors such as glyceryl trinitrate (GTN) and AS would be beneficial to M2 macrophage shift and could reduce cytokines that are associated with or precede atherosclerosis and thus may be useful therapeutic strategies for atherosclerosis.

*(7) Inhibitors of the Microsomal Triglyceride Transfer Protein (MTP)*. Inhibitors of the microsomal triglyceride transfer protein (MTP) have been found to downregulate apolipoprotein B- (ApoB-) containing lipoproteins in animals and humans effectively. A single-arm, open-label, phase 3 study of lomitapide, an inhibitor of MTP, was down for treatment of patients with homozygous familial hypercholesterolemia [164]. The study concluded that lomitapide could be favorable to reduce the levels of LDL cholesterol and ApoB in adult patients with homozygous familial hypercholesterolemia. Hewing et al. suggested that in MTP inhibitor-treated mice, M2 markers including arginase-I and MR were increased compared to those in the control group [165]. We consider whether the lipid-lowering effect of MTP inhibitors attributes to M2 macrophage shift. The clear mechanism of inhibitors of MTP is urgently needed to be explored, and lomitapide could be a promising compound for atherosclerosis treatment.

*(8) Conjugated Linoleic Acid (CLA)*. Conjugated linoleic acid (CLA) which was first found to inhibit chemically induced cancer has been reported to regress atherosclerosis plaques by some researchers [166–168]. McCarthy et al. indicated further that CLA supplementation increased IL-10 expression and induced macrophages to skew to an anti-inflammatory M2 phenotype in vitro or in vivo [169]. In addition, CLA can act as an agonist of PPARs and is involved in modulating inflammation. Bruen et al. recently reported that CLA reduced the level of the M1 macrophage marker CD68 and alleviated some M2 markers including CD163 and mannose receptors in human peripheral blood mononuclear cell-derived macrophages [170]. Besides, they confirmed that CLA could limit foam cell formation, reduce inflammation mediators, and affect atherosclerosis lesion development. This suggest CLA as a potential compound for atherosclerosis treatment.

*(9) Erythropoietin (EPO), Helix B Surface Peptide (HBSP), and Hemopexin (Hx)*. Helix B surface peptide (HBSP) is a nonerythropoietic, tissue-protective compound derived from EPO. Studies showed that HBSP as well as EPO have a protective effect on atherosclerosis. Ueba et al. demonstrated that HBSP significantly downregulated M1 macrophages and the M1/M2 ratio while it increased the level of IL-10 in coronary atherosclerotic lesions, suggesting that HBSP might promote macrophages to a predominant M2 phenotype [171]. A study explained that the receptors of EPO, receptors of colony-stimulating factor (CSF), and target cells for erythropoietin and colony-stimulating factor have something in common [172]. We speculate that EPO may exert an impact on macrophages like M-CSF which polarizes macrophages to an M2 phenotype. In addition, it was reported by Mehta et al. that ApoE^−/−^ mice lacking hemopexin (Hx) intensified the formation of atherosclerosis via inducing oxidative stress and regulating macrophage function while Hx and ApoE double-knockout (HxE^−/−^) mice with human Hx injection showed a shift from M1 to M2 macrophages and inhibited the progression of atherosclerosis in ApoE^−/−^ mice [173]. Thus, it is also possible for EPO to affect macrophage polarity through modulating the synthesis of Hx.

*(10) Pomegranate Juice and Polyphenols*. In the evidence of the anti-inflammation function of pomegranate, Aharoni et al. investigated the association of pomegranate juice (PJ) as well as its polyphenols and macrophage phenotypes [174]. They reported that the secretion of IL-10 was promoted by PJ and polyphenols in a dose-dependent manner. Mice supplemented with PJ, comparing to the control group supplemented with water, showed a 36% decrease and a 41% decrease in TNF*α* secretion and IL-6 secretion, respectively, indicating a macrophage shift towards the M2 phenotype. Besides, ApoE-KO mice treated with PJ decreased the progressive inflammation in the aorta atherosclerotic lesion with aging. The study suggests that PJ or its polyphenols may result in macrophage polarization towards the M2 phenotype and lead to antiatherosclerosis effect.

A growing body of evidence demonstrates that an altered phenotype of macrophages towards M2 macrophages is associated with the progression of atherosclerosis. However, most of them are developed in cell or animal experiment levels. No clinical study has been carried on regarding M2 polarization therapy, which means further researches are needed. As to the reported targets, a major part is associated with the signaling of M2 polarization pathways, especially p-STAT6 and PPAR*γ* activation. Thiazolidinediones (TZDs) such as rosiglitazone and thiazolidinedione, well-known medicines for diabetes mellitus therapy, are reported to polarize macrophages towards an M2 phenotype, which therefore benefits atherosclerosis reverse. Future studies will be focused on further characterizing M2 polarization pathways and exploring new drugs for M2 polarization or the potential macrophage shifting effect of existing drugs, in an effort to find novel effective treatment for atherosclerosis.

## 6. Update of Macrophage Classification

Influenced by different environmental signals, macrophages can undergo different polarizations and play diverse roles in the pathogenesis of many conditions. More different phenotypes of macrophages were found, and the latest macrophage classification of phenotypes includes M1, M2, M4, Mox, HA-mac, M (Hb), and Mhem [30, 49]. M1 macrophages, playing an important role in atherosclerosis development, were also known as proinflammatory macrophages which could produce IL-6, IL-12, and TGF-*α*. M2 phenotypes were divided into four subgroups including M2a, M2b, M2c, and M2d, induced by IL-4/IL-13, immune complexes, TGF-*β*/IL-10/glucocorticoids, and TLR+A2R (adenosine A2A receptor) ligands, respectively. M2a macrophages, known as “wound-healing macrophages,” express high levels of the mannose receptor (MR or CD206) and secrete profibrotic factors such as fibronectin, insulin-like growth factor (IGF), and transforming growth factor *β* (TGF-*β*) contributing to the tissue repair [175]. The M2a phenotype, on the other way, shows potent anti-inflammatory properties and is characterized by poor phagocytic ability and suppression of proinflammatory cytokine release. M2d macrophages, producing high levels of IL-10 and vascular endothelial growth factor (VEGF) and low levels of TNF and IL-12, were also thought to have a potential role in tissue repair. Different with other phenotypes of M2 macrophages, M2d macrophages do not express Ym1, Fizz1, or CD206. According to the tissue repairing potential and anti-inflammation product expression, M2a and M2d might be critical on the course of atherosclerosis regression. M2b and M2c share regulatory functions and are referred to as “regulatory macrophages.” They express high levels of IL-10 and the Mer receptor tyrosine kinase (MerTK) providing them with high efferocytosis capacity which plays an important role in anti-atherosclerosis formation [13]. Noticeably, M2b macrophages retain the ability of producing high levels of proinflammatory cytokines including IL-1*β*, IL-6, and TNF and low IL-12 expression [19, 32]. Although significant progress has been made in characterizing the phenotype and functions of the M2 subtypes, several questions are still open. How are the four subtypes involved in atherosclerosis plaque development and regression process? How to promote atherosclerosis diminution through regulating M2 subtypes polarization? Much remains to be discovered. The M4 macrophage was specifically induced by chemokine CXCL4 and could secrete proinflammatory molecules, such as IL-6 and TNF*α*. Mox macrophages were shown to be only presented in murine and promote atherosclerotic plaque progression through increasing the level of IL-1*β* and ROS. HA-mac was found in hemorrhagic lesions of human plaques and expressed high levels of CD163 via which Hb–Hp complexes could be tested and then cleared and oxidative stress could be reduced. In addition, the HA-mac phenotype could produce anti-inflammatory IL-10 and possesses atherosclerosis protective effect. Besides, Mhem and M (Hb) phenotypes were also found beneficial for antiatherosclerotic plaques, especially for reducing intraplaque hemorrhages. With the development of scientific progress and experimental techniques, more macrophage phenotypes and their biological characteristics might be found. However, the M2 phenotype remains the most potential target for antiatherosclerosis therapy.

## 7. Conclusion and Prospects

Atherosclerosis is a chronic inflammation disease in which macrophages are involved. Although statins are widely used for atherosclerotic plaque regression as well as LDL lowering, more efficient novel drugs or adjuvant medicines are still needed. Many compelling researches have indicated that M2 macrophages play a critical role in atherosclerosis regression. Some compounds or biomolecules have showed that they could lead to macrophage shift towards the M2 phenotype, thus generating an anti-atherosclerosis effect. Thereby, M2 macrophages may be a potential target for atherosclerosis treatment. Recently, few of the compounds, such as rosiglitazone and thiazolidinedione, were tested in patients. However, many of them are still under laboratory studies and tests in animal models or patients of AS are needed. Besides, more researches about the detailed mechanism and further studies on the above compounds may promote the development of antiatherosclerosis therapy.

## Figures and Tables

**Table 1:** Marker molecules of M2 macrophages.

	M2 marker molecules
Surface markers	MR/CD206, SR-A, MHCII, CD86, CD163, IL-R*α*
Enzymes	Arg1, Ym1, Fizz1, MMP12, MMP7, MERTK
Secretions	IL-10, IL-6, TGF-*β*, IL-12^low^, CCL17, CCL22, CCL18, PTX3, AMAC1, TNF*α*^low^, VEGF

**Table 2:** Enzymes as therapeutic targets for M2 polarization.

Targets	Way to affect the targets	Experiment animals or cells	Potential mechanism	Compounds or medicine	References
DPP	-	Mononuclear cells obtained from humans; ApoE^−/−^ mice	Via the SDF-1/CXCR4 signaling	Gliptins and Sitagliptin	[90, 91, 93]
HDAC9	-	LDLR^−/−^ and LDLR^−/−^HDAC9^−/−^ mice	Increased accumulation of total acetylated H3 and H3K9 by the promoters of ABCA1, ABCG1, and PPAR*γ* in macrophages		[94]
PKA	+	RAW267.4 macrophage cells			[79]
CHIT1	+	Mouse macrophage cells			[96]
GSK3*α*	-		Increased P-STAT6		[97]
ATGL	-	ATGL^−/−^ mice	Insufficient lipolysis influenced macrophage polarization to an M2 phenotype		[98]
NAMPT	-	Patients with coronary artery disease (CAD); murine bone marrow-derived macrophages	Inhibited M1 polarization in macrophages; enhance the expression of CD163 and PPAR	FK866	[99, 100]

DPP: dipeptidyl peptidase; HDAC9: histone deacetylase; PKA: protein kinase A; CHIT1: chitinase 1; GSK3*α*: glycogen synthase kinase; ATGL: adipose triglyceride lipase; NAMPT: nicotinamide phosphoribosyl transferase; ATGL^−/−^ mice: adipose triglyceride lipase-deficient mice; LDLR^−/−^ mice: LDL receptor-deficient mice; LDLR^−/−^HDAC9^−/−^ mice: LDL receptor and HDAC9 double-deficient mice. +: activate or upregulate the targets; -: inhibit or downregulate the targets.

**Table 3:** Receptors as potential targets for M2 polarization.

Targets	Way to affect the targets	Experiment animals or cells	Effect	Compounds or medicine	References
SR-A	+	SR-A^−/−^ mice	Lack of SR-A promotes M1 polarization by activating NF-*κ*B and suppressing STAT6 signaling		[102–105]
Notch1R	-	THP-1 cells treated with Notch1R siRNA	Enhanced M2 macrophage activation and upregulated anti-inflammatory cytokine secretion	DAPT	[106]
Fc*γ*R	-	ApoE^−/−^Fc*γ*RIIb^−/−^ mice	Upregulated Arg1 and lower iNOS expression than ApoE^−/−^ mice		[107, 108]
Nr1D1	+	Rev-erba knockdown mice	Macrophages obtained from Rev-erba knockdown mice present lower M2 while overexpression of Rev-erba increased the expression of M2 markers. Heme promoted M2 marker expression	Heme	[111]
Sdc-1	+	Sdc-1^+/+^ and Sdc-1^−/−^ macrophages	Contributed to the motility that specifically induced M2 macrophage populations		[112]
KCa3.1	-	Human monocytes; ApoE^−/−^ mice on a C57BL/6 background	Reduced plaque rupture and luminal thrombus in carotid arteries, decrease expression of M1 markers, and enhance expression of M2 markers within atherosclerotic lesion	TRAM-34	[114]

SR-A: class A scavenger receptor; SR-A^−/−^ mice: SR-A-deficient mice; Notgh1R: Notch1 receptor; THP-1 cells: a human monocytic cell line; Fc*γ*R: Fc*γ* receptors; iNOS: inducible nitric oxide synthase; Nr1D1: Rev-erba; Sdc-1: Syndecan-1; KCa3.1: calcium-activated potassium channel; DAPT: N-[N-(3,5-difluorophenacetyl)-l-alanyl]-S-phenylglycine t-butyl ester, a *γ*-secretase inhibitor; Sdc-1^+/+^: wild-type macrophages; Sdc-1^−/−^ macrophages: Sdc-1-deficient macrophages.

**Table 4:** Transcription factors as potential targets for M2 polarization.

Targets	Way to affect the targets	Experiment animals or cells	Effect	Compounds or medicine	References
PPAR*γ*	+	Macrophages in human carotid atherosclerotic lesions	Increased the expression of the anti-inflammatory M2 cytokine Arg1 and attenuated the iNOS/Arg1 ratio	Thiazolidinediones (TZDs) such as rosiglitazone and thiazolidinedione	[121, 122]
PPAR*δ*	+	C57BL/6 LDLR^−/−^ mice	Upregulated M2 cytokines, while decreasing the expression of M1 cytokines	GW1516	[123]
NOR1	+	Bone marrow-derived macrophages obtained from C57BL/6J mice	A direct target of STAT6 and then promoted M2 expression		[124]
KLF4	+	Mouse peritoneal macrophages; myeloid KLF4-deficient mice	Promoted M2 marker expression by cooperating with STAT6; related with the expression of PPAR*γ*, thus regulating M2 polarization	Kallistatin	[70, 125]
FoxO	+	Myeloid FoxO1^−/−^ mice	Increase IL-10 gene expression and decrease Akt phosphorylation in FoxO-deficient mice; Pdk1-FoxO1 pathway was suggested		[130]
TLE1	+	Human peripheral blood mononuclear cells	M2 markers including TGF-*β* and IL-10 were observed decreasing when TLE1 was silenced by siRNA		[132]

NOR1: the neuron-derived orphan receptor 1; FoxO: forkhead transcription factors; TLE1: transducin-like enhancer of split-1; myeloid FoxO1^−/−^ mice: generated by crossing FoxO1fl/fl mice with LysMCre mice.

**Table 5:** Other molecules promoting M2 polarization.

Biomolecules	Experiment animal/cells	Effect	References
27-OH	PBMC from healthy donors	Increased the secretion of IL-10 and expression of LXR and ABCA1	[134]
VD	Hypercholesterolemic swine	Affected the process of M2 polarization; decreased the 27-hydroxycholesterol level	[136]
Adiponectin	Human peripheral blood monocytes	Decreased adiponectin was related with lower levels of M2 markers such as IL-10 and arginase-1 in the liver of mice	[144, 145, 147]
Inhibitors of Sema3E	Macrophages of advanced atherosclerotic lesions of ApoE^–/–^ mice	Inhibiting Sema3E may promote M2 polarization and downregulate the inflammation in atherosclerotic lesions	[152]
BMP-7	ApoE^−/−^ mice: sham, PLCA, and PLCA+ BMP-7	Upregulated BMP-7R expression which led to activation of PI3K, Akt, and mTOR, thus enhancing M2 macrophage polarization	[153]
Trx-1	Murine peritoneal and human macrophages	Elevated M2 markers including CD206 and IL-10	[154]
ApoA-I	ApoA-I^−/−^ or apolipoprotein E-deficient mice	Increased M2 macrophage markers and decreased M1 macrophages in atherosclerotic plaques	[156, 159]
HNO donors: AS or GTN	C57/Bl6 mice; human monocytes	Increased CD200R and CD206 expression and mRNA gene expression of CD206 and the anti-inflammatory gene SR-B1 in macrophages treated with IL-4 coincubated with AS	[163]
Inhibitors of MTP	LDLR^−/−^ mice	Increased M2 markers including arginase-I and MR	[165]
CLA	ApoE^−/−^ C57BL/6J mouse; bone marrow-derived macrophages	Reduced the level of CD68 and alleviate the levels of CD163 and mannose receptor in human macrophages	[166, 169, 170]
EPO	WHHLMI rabbits; HUVECs	Reduced HUVEC apoptosis and THP-1 production of TNF*α* and MMP-9; activated Akt and ERK1/2; decreased M1 macrophages and the M1/M2 ratio; increased expression of IL-10 in coronary atherosclerotic lesions	[171, 172]
HBSP			
Hx	HxE^−/−^ mice	HxE^−/−^ mice with human Hx injection showed a shift from M1 to M2 macrophages and inhibited the progression of atherosclerosis in ApoE^−/−^ mice	[173]
PJ and polyphenols	ApoE^−/−^ mice and mouse peritoneal macrophages	Promoted the expression of IL-10 and decreased the secretion of TNF*α* and IL-6	[174]

27-OH: 27-hydroxycholesterol; PBMC: peripheral blood mononuclear cells; VD: vitamin D; Sema3E: Semaphorin 3E; BMP-7: bone morphogenetic protein-7; Trx: thioredoxin; ApoA-I: apolipoprotein A-I; HNO donors: nitroxyl anion donors; MTP: microsomal triglyceride transfer protein; CLA: conjugated linoleic acid; EPO: erythropoietin; HBSP: helix B surface peptide; Hx: hemopexin; PLCA: partial left carotid artery ligation; AS: Angeli's salt; GTN: glyceryl trinitrate; HUVECs: human umbilical vein endothelial cells; HxE^−/−^ mice: Hx and ApoE double-knockout mice; PJ: pomegranate juice.
